# Supplementary material for: Transcriptome Analysis Reveals Vimentin-Induced Disruption of Cell–Cell Associations Augments Breast Cancer Cell Migration
Source: Cells. 2022 Dec 13;11(24):4035. doi: 10.3390/cells11244035 (PMC9776984; doi:10.3390/cells11244035)
Supplement: Supplementary file 1 [file cells-11-04035-s001.zip › DEGs and enrichment analysis.zip!/DEGs and enrichment analysis/WT_VIMvsVector KEGG/ALL/WT_VIMvsVector_ALL.pathway_map.zip!/WT_VIMvsVector_ALL.pathway_map/src/hsa05034.html]

hsa05034


- hsa:7054

- Up regulated genes

ENSG00000180176(2.50876120874943)

- hsa:7054

- Up regulated genes

ENSG00000180176(2.50876120874943)

- hsa:7054

- Up regulated genes

ENSG00000180176(2.50876120874943)

- hsa:1388

- Up regulated genes

novel.600(2.78845209317737)
- hsa:90993

- Down regulated genes

ENSG00000157613(-3.21711806186639)

- hsa:1388

- Up regulated genes

novel.600(2.78845209317737)
- hsa:90993

- Down regulated genes

ENSG00000157613(-3.21711806186639)

- hsa:51764

- Up regulated genes

ENSG00000127588(3.22298284123795)

- hsa:8338

- Up regulated genes

ENSG00000184260(2.31199468222276)
- hsa:8336

- Up regulated genes

ENSG00000218690(2.22495124008211)

- hsa:8970

- Up regulated genes

ENSG00000124635(1.92753744639879)

Close
